# Adenovirus Encoded Adjuvant (AdEnA) anti-CTLA-4, a novel strategy to improve Adenovirus based vaccines against infectious diseases and cancer

**DOI:** 10.3389/fimmu.2023.1156714

**Published:** 2023-04-26

**Authors:** Anna Morena D’Alise, Linda Nocchi, Irene Garzia, Laura Seclì, Luigia Infante, Fulvia Troise, Gabriella Cotugno, Simona Allocca, Giuseppina Romano, Armin Lahm, Guido Leoni, Emanuele Sasso, Elisa Scarselli, Alfredo Nicosia

**Affiliations:** ^1^ Nouscom Srl, Rome, Italy; ^2^ Department of Biology, University of Rome “Tor Vergata”, Rome, Italy; ^3^ Department of Molecular Medicine and Medical Biotechnology, University of Naples Federico II, Naples, Italy; ^4^ CEINGE-Advanced Biotechnologies s.c. a.r.l., Naples, Italy

**Keywords:** vaccine, genetic adjuvants, immune response, cancer, infectious disease, neoantigens

## Abstract

**Introduction:**

Virus vectored genetic vaccines (Vvgv) represent a promising approach for eliciting immune protection against infectious diseases and cancer. However, at variance with classical vaccines to date, no adjuvant has been combined with clinically approved genetic vaccines, possibly due to the detrimental effect of the adjuvant-induced innate response on the expression driven by the genetic vaccine vector. We reasoned that a potential novel approach to develop adjuvants for genetic vaccines would be to “synchronize” in time and space the activity of the adjuvant with that of the vaccine.

**Methods:**

To this aim, we generated an Adenovirus vector encoding a murine anti-CTLA-4 monoclonal antibody (Ad-9D9) as a genetic adjuvant for Adenovirus based vaccines.

**Results:**

The co-delivery of Ad-9D9 with an Adeno-based COVID-19 vaccine encoding the Spike protein resulted in stronger cellular and humoral immune responses. In contrast, only a modest adjuvant effect was achieved when combining the vaccine with the same anti-CTLA-4 in its proteinaceous form. Importantly, the administration of the adjuvant vector at different sites of the vaccine vector abrogates the immunostimulatory effect. We showed that the adjuvant activity of Ad-α-CTLA-4 is independent from the vaccine antigen as it improved the immune response and efficacy of an Adenovirus based polyepitope vaccine encoding tumor neoantigens.

**Discussion:**

Our study demonstrated that the combination of Adenovirus Encoded Adjuvant (AdEnA) with an Adeno-encoded antigen vaccine enhances immune responses to viral and tumor antigens, representing a potent approach to develop more effective genetic vaccines.

## Introduction

With the approval of the Ebola vaccine *Ervebo* (based on live attenuated recombinant based on live attenuated recombinant vesicular stomatitis virus, VSV) and the anti-COVID-19 vaccines *INN-Ad26.COV2-S* and *Vaxzevria* (based on replication incompetent Adenoviral vectors), virus vectored genetic vaccines (Vvgv) have entered the armory of vaccine technologies against infectious diseases and hold the promise for novel therapeutic approaches against cancer (1–3). While displaying a number of advantages over classical vaccines based on recombinant proteins, such as the ease and reproducibility of the production process, and the intrinsic ability to induce cellular immunity (4), Vvgv are still a newborn technology and have not been optimized, yet. The goal of an effective vaccination is to elicit robust humoral and cellular immune responses capable of affording protection from disease agents. To this end, vaccine adjuvants were shown to play a fundamental role in increasing vaccine immunological potency and protection. This is achieved by combined administration of vaccine and immune-modulatory molecules, as it has been well-documented, and found to have broad applications spanning from infectious diseases to cancer (5–10). Classical vaccines can take advantage from the use of adjuvant compounds, such as aluminum salts, oil in water emulsion, and the more recently approved AS01, AS03, AS04, CpG ODN, and MF59, as a mean to improve magnitude and quality of antigen-specific adaptive immunity for optimized protection against pathogens (11, 12). In contrast, Vvgv are relying only on the vehicle used for their transduction in the target cells and, to date, there is no adjuvant being used in the formulation of clinically approved Vvgv. Failure to combine known adjuvants to Vvgv is most likely due to the detrimental effect of the adjuvant-induced innate response on the level and duration of antigen expression driven by the genetic vaccine vector (13). Nevertheless, plasmid encoded immunomodulator molecules were shown to improve DNA vaccines in a number of animal models (14). Vvgv-induced adaptive immunity is orchestrated by a distinct sequence of signals and cellular events that must occur in the right place and at the right time to achieve optimal response. We reasoned that a potential novel approach to develop adjuvants for Vvgv would be to ‘synchronize’ in time and space the activity of the adjuvant with that of the vaccine. To achieve this goal, genes encoding for immunomodulatory molecules instead of the adjuvant itself, should be co-delivered using the same vector-based system of the genetic vaccine, to ensure local and timely expression of the immunomodulator together with the antigen. As a proof of concept of this strategy, we generated an Adenovirus vector encoding a murine anti-CTLA-4 monoclonal antibody (Ad-9D9), and tested its adjuvant effect on adaptive immune responses when co-administered in a mixture with Adenoviral-based vaccines encoding for viral or cancer antigens. The rationale behind the choice of anti-CTLA-4 relies on the known role of CTLA-4 in restraining T-cell activation. Upon TCR ligation, CTLA-4 is upregulated and outcompetes CD28 for B7 ligand binding, limiting positive co-stimulation by CD28 (15). The modulation of CTLA-4 mediated T-cell inhibition holds great promise in several clinical applications. Moreover, it has been largely demonstrated that CTLA-4 blockade results in enhanced immune responses as well as improved anti-tumor activity (16–20). We showed a potent enhancement of both cellular and humoral Sars-CoV-2 Adenovirus vaccine-induced immune responses when co-administered with Ad-9D9 in mice, as well as an increase in dominant and sub-dominant tumor neoantigen-specific immunity. The same effect was not achieved by using α-CTLA-4 in its protein form. The co-administration of the vaccine and the genetic adjuvant at the same time and in the same anatomical site is required for the adjuvant effect. In an advanced tumor setting, a strong anti-tumor effect was obtained combining an Adenovirus vector encoding tumor neoantigens, the Ad-9D9 and anti-PD-1 monoclonal antibody. The contribute of the encoded antibody to the anti-tumor efficacy correlated with the reduction of intratumoral Treg, a significant increase of cytotoxic T effector cells, and the development of a long-lasting protective CD8 T cell memory. Importantly, all these effects were obtained with an amount of vector-produced antibody released in the circulation far lower than that observed when the same antibody was administered in the form of protein, potentially reducing the toxicity of the genetic adjuvant while preserving a strong adjuvant effect.

## Materials and methods

### Mice

Six-week-old female BALB/c or C57BL/6 mice were purchased from Envigo. Mouse colony management and all day-to-day care of the experimental animals were performed by trained mouse house staff at Plaisant (Castel Romano, Italy). Six-week-old female immunocompetent chimeric mice engineered to express the human CTLA-4 (HuGEMM, CrownBio) in C57Bl/6 background were provided by and housed at CrownBio. All the *in vivo* experimental procedures were approved by the local animal ethics council and performed in accordance with national and international laws and policies (EEC Council Directive 86/609; Italian Legislative Decree 26/14). The ethical committee of the Italian Ministry of Health approved this research.

### Cell culture

CT26 (Balb/c murine colon carcinoma) and MC38 (C57Bl/6 murine colon adenocarcinoma) cell lines were purchased from ATCC. Cells were cultured in complete RPMI-1640 and DMEM, respectively, supplemented with 10% fetal bovine serum, 2mM L-glutamine, 1% (v/v) penicillin/streptomycin and maintained at 37°C in 5% CO_2_. CT26 cells were used on their passage 7-9; MC38 cells were used on passage 37. Both cell lines were tested for the absence of mycoplasma contamination by PCR.

### *In vitro* infection

HeLa cells were infected with Ad-9D9 (murine anti-CTLA-4) and Ad-Ipi (human anti-CTLA-4 Ipilimumab) at Multiplicity of infection (MOI) 100 and supernatant was collected after 48h for measuring 9D9 and Ipilimumab levels by Elisa and Western Blot.

### Adenoviral vectors production

Adenoviral vectors were generated as previously described (21). The amino acid sequence of the 9D9 heavy and light chain variable domains were extracted from published sequences (US9868961B2). The sequence of the heavy chain variable domain sequence was modified according to “mod #4”, as described in (22). The complete 9D9 construct was then assembled identical to the construct shown in (22): a mouse VH signal sequence followed by the 9D9 VH domain, then the mouse IgG2A heavy chain constant region followed by a RGRKRRS cleavage site and a SGS linker, followed by a P2A peptide sequence, a mouse kappa light chain signal sequence, the 9D9 VL domain and the mouse kappa light chain constant domain. The complete encoded amino acid sequence is reported in **Supplementary Material**.

### Serum collection

Mouse blood was collected from the submandibular vein before any treatment and then at 24 hours, 72 hours, 7 days, 10 days and 14 days post injection. Serum was isolated by centrifugation at 12500g for 5 minutes at room temperature and stored at -20°C until use. Serum samples were used for Elisa assay, to measure anti-CTLA-4 levels.

### Anti-CTLA-4 9D9 Elisa assay

Recombinant murine CTLA-4 (Sino Biological) or human CTLA-4 (Sino Biological) were incubated at 5µg/ml on a 96-well plate (Nunc Maxisorp) overnight at 4°C. After washing with PBS-tween 0.05% and blocking in BSA 5%, supernatant or serum samples (1:50 and 1:10 diluted, respectively in PBS+BSA 2.5%) and standard curve were incubated for 90 minutes at room temperature. For standard curve, 9D9_2A (Invivogen) or Ipilimumab (kindly gifted by Prof. Ascierto, Pascale Institute, Naples) were used serially diluted in PBS+BSA 2.5%. Anti-mouse IgG Fc-specific (Sigma) and anti-human IgG Fab-specific (Sigma) alkaline-phosphatase conjugate secondary antibodies were used for 9D9 and Ipi detection, respectively. Para-Nitrophenylphosphate (PNPP) detection substrate (Sigma) was used for the detection and optical density (OD) absorbance was read at 405nm (620nm absorbance was used as background) using Tecan plate reader. For data analysis, Four Parameter Logistic Regression was used.

### Anti-spike Elisa

His-tagged Sars-CoV-2 Spike Protein (Sino Biological) was incubated on a 96-well plate at the concentration of 2.5 µg/ml, overnight at 4°C. After washing with PBS-tween 0.05% and blocking with Milk 3% in PBS, plate was incubated with serially diluted serum samples, starting from 1:100 up to 1:24300 in milk 1%-PBS-tween. Sars-Cov2 (COVID-19) spike antibody (IgG1) [1A9] (GeneTex) was used as positive control, at 1:50 and 1:250 dilution. Binding was detected with anti-mouse IgG Fc-specific secondary antibody (Sigma), 1:10000 diluted in milk 1%-PBS-tween. Alkaline phosphatase substrate PNPP was used. Absorbance was read at 405 and 620 nm using Tecan plate reader. The end point titer was defined as the highest serum dilution that resulted in an absorbance value [OD (optical density)] just above the calculated background of 3-fold the OD from a naïve pre-immunized mice.

### Western blot

Hela cells were infected with Ad-encoded anti-CTLA-4 at MOI 100. Forty-eight hours post infection, supernatant was collected. Protein concentration was determined by the Bradford assay. Proteins were separated by SDS-PAGE under reducing conditions using NuPAGE^®^ 4-12% Bis-Tris Gradient gels (Invitrogen) and PVDF membranes were blotted at room temperature for 1 hour with the appropriate antibodies: anti human IgG Heavy + Light horseradish peroxidase (HRP) conjugated 1:2500 (Promega) and goat Anti mouse IgG (Fc specific) HRP conjugated 1:2000 (Sigma). As secondary antibody, goat Anti-Rabbit IgG (whole molecule) 1:2000 HRP conjugated (Sigma) was used. Detection was performed using Electrochemiluminescence (ECL) detection reagent (Pierce).

### *In vivo* tumor growth

For established tumor setting experiments, 2x10^6^ CT26 cells and 2x10^5^ MC38 cells were subcutaneously (SC) injected into the right flank of Balb/c and C57Bl/6 mice, respectively. After 5-7 days, tumor mass was measured with a digital caliper, applying the formula: 0.5 × length × width^2^, where the length is the longer dimension. Animals were then randomized based on their tumor size (tumor size average per group 30-70 mm^3^) and treatments began (day 0). Tumor growth was measured every 3–4 days and mice were euthanized as soon as signs of distress or a tumor volume above 1500 mm^3^ was reached. For second tumor challenge, tumor free mice were inoculated with 2x10^5^ CT26 cells on day 42 post the first challenge.

### *In vivo* treatments

Adenoviral vaccines encoding for Spike (Adeno26) or tumor neoantigens (GAd) were administered *via* intramuscular (IM) injection in both quadriceps by delivering a volume of 50 μl per side at 10^8^ viral particles (vp) or 2x10^7^ vp. Ad-9D9 and Ad-Ipi were administered IM at 10^8^ vp. When Ad-9D9 vector was mixed with the vaccine, 50 μl of the mix were delivered per side. Anti-CTLA-4 in its protein form (Invivogen, clone 9D9 isotype 2A) was administered SC at 33 µg/mouse, close to the injection site of the vaccine. Ipilimumab was injected intraperitoneal (IP) at 100 µg/mouse. For efficacy studies, α-mPD-1 (BioXcell, clone RMP114) was administered IP, at a dosage of 200 μg twice a week, starting from day 0 until day 17. Adenovirus-based vaccines were prepared in A195 buffer (pH 7.4; 10mM Trizma, 10 mM Histidine, 5% sucrose, 75 mM NaCl, 1 mM MgCl_2_, 0.02% PS-80, 0.1 mM EDTA, 0.5% (v/v) Ethanol) or A438 buffer (pH 6.6; 10 mM Histidine; 7.5% sucrose; 35 mM NaCl; 1 mM MgCl2; 0.1% Polysorbate (PS)-80; 0.1 mM EDTA; 0.5% (v/v) Ethanol.

### *Ex vivo* immune analysis

IFN-γ ELISpot assay was performed 14 days post immunization, on cell suspension of spleens. MSIP S4510 plates (Millipore) were coated with 10 μg/ml of anti-mouse IFN-γ antibody (U-CyTech) and incubated overnight at 4°C. After washing and blocking, freshly isolated mouse splenocytes were plated in duplicate at two different cell densities (5x10^5^ and 2.5x10^5^ cells) and stimulated overnight with peptide pools or single peptides at a final concentration of 1 μg/ml. The peptide diluent dimethyl sulfoxide (DMSO) and Concanavalin A (ConA) were used as negative and positive controls, respectively. Plates were then incubated with biotinylated anti-mouse IFN-γ antibody (dilution: 1/100; U-CyTech), conjugated streptavidin–alkaline phosphatase (dilution: 1/2500; BD Biosciences) and finally with 5-bromo-4-chloro-3-indoyl-phosphate/nitro blue tetrazolium 1-Step solution (Thermo Fisher Scientific). An automated enzyme linked immunosorbent–spot assay video analysis system automated plate reader was used to analyze plates. ELISpot data were expressed as IFN-γ spot forming cells (SFCs) per million of splenocytes. ELISpot responses were considered positive if all the following conditions occurred: (i) IFN-γ production present in ConA stimulated wells, (ii) the number of spots seen in positive wells was three times the number obtained in the negative control wells (dimethyl sulfoxide), (iii) at least 30 specific spots/million splenocytes.

### Cell suspension from iliac lymph nodes, spleen, blood and tumor

Iliac lymph nodes were collected 9 days post immunization with GAd vaccine or GAd vaccine + Ad-9D9 and immediately processed to obtain a cell suspension, by tearing and gentle scraping of the organ. Cell suspension was then incubated with Collagenase IV (0.4 mg/ml) and DNAse I (0.2 mg/ml) for 30 minutes at 37°C, in agitation, followed by staining for surface markers and FACS analysis.

Spleens for IFN-γ ELISpot were collected 14 days post immunization. Splenocytes were isolated by manual pressing of the organ with a piston, followed by lysis of red blood cells by incubation with ACK buffer. Finally, cells were filtered through a 70-µm cell strainer, ready for the next analysis.

Blood for staining and FACS analysis was collected 14 days post immunization in heparin-containing tubes, incubated with ACK buffer for 2-3 times for 10 minutes each, centrifuged at 1500 rpm for 5 minutes, filtered through 70-µm cell strainer, and ready for the staining and FACS analysis.

Tumors were dissected out 9 days post immunization, dissociated and digested with Miltenyi Tumor Dissociation kit, following manufacturer’s instructions. Tumor homogenates were then filtered through a 70-µm cell strainer to generate single-cell suspension, ready for staining with markers of interest.

### Immune staining of lymph nodes, blood and tumor infiltrating lymphocytes

Cell suspensions from iliac lymph nodes, blood and tumors were first incubated with 1µg/10^6^ cells of Fc block treatment (BD Pharmigen) and then stained for surface markers in FACS buffer (PBS + 1% FBS). For Treg staining on cells isolated from iliac lymph nodes: Live/Dead (Fixable Near-IR Dead Cell stain kit, Life Technologies); CD3 FITC, clone 145-2C11 (Biolegend); CD4 Pacific Blue, clone RM4-5 (Biolegend); Foxp3 APC, clone FJK-16S (eBioscience). For Treg staining on cells isolated from tumors: Live/Dead (Fixable Near-IR Dead Cell stain kit, Life Technologies); CD45 PerCP-Cyanine 5.5 (eBioscience); CD4 Pacific Blue, (Biolegend); CD8 Brilliant Violet 510, clone 53-6.7 (Biolegend); Foxp3 APC, (eBioscience). For effector memory staining on cells isolated from blood: Live/Dead (Fixable Near-IR); CD8 PerCP, (Biolegend); CD44 APC (Biolegend); CD62L FITC, clone MEL-14 (BD Pharmigen).

### Statistical analyses

Statistical significance was determined by GraphPad Prism (version 9) using the nonparametric, two-tailed Mann-Whitney U test or as otherwise stated in the figure legend.

## Results

### Adeno encoded anti-CTLA-4 (Ad-9D9) enhances cellular and humoral response to Sars-Cov2 Adeno based vaccine

**Figure 1:**
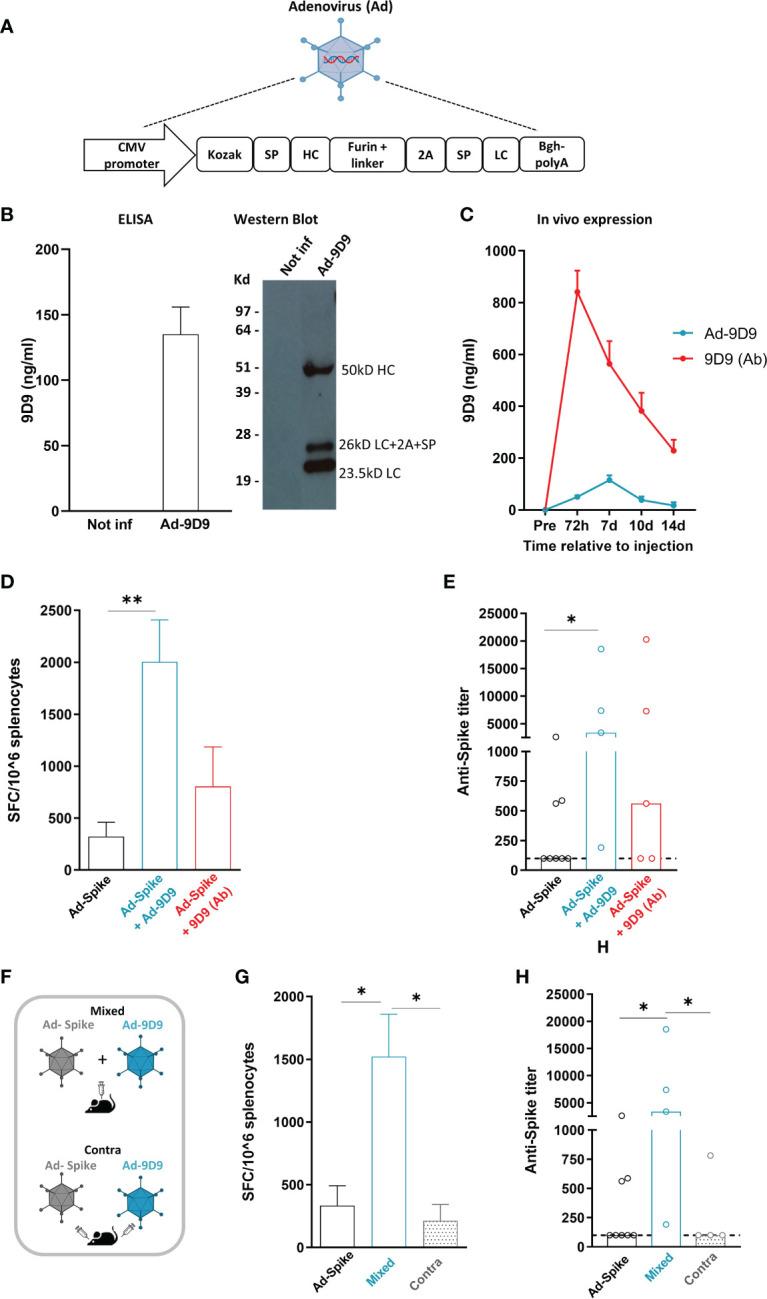
Adeno encoded anti-CTLA-4 enhances cellular and humoral response to Sars-Cov2 Adeno based vaccine when co-administered with the vaccine **(A)** Anti-mouse CTLA-4 antibody (Ad-9D9) gene sequence was inserted in a replication defective Adenovirus 6. SP = Signal peptide; HC = Heavy chain; LC = Light chain. **(B)** *In vitro* expression of 9D9 in HeLa cells infected with Ad-9D9 at 100 MOI (Multiplicity of Infection) was assessed by Elisa and Western Blot on supernatant collected 48 hours post infection. *In vitro* experiments were performed in triplicate. **(C)** *In vivo* expression of 9D9 measured by ELISA on sera from C57Bl/6 mice injected with Ad-9D9 (10^8^ vp, IM; blue curve; n=5) or 9D9 antibody (Ab, 33 µg, SC; red curve; n=5). Mean ± SEM is shown. **(D)** T cell response to Spike peptide stimulation measured by ex vivo IFN-ɣ ELISpot assay, 14 days after IM injection of Ad26-Spike vaccine alone or in combination with Ad-9D9 or 9D9 (Ab, SC injected). ** p <0.005, n = 5-8. Mean ± SEM is shown. **(E)** Spike Sars-Cov2–specific IgG titers in the serum collected 14 days after vaccination, measured by ELISA on recombinant full-length spike protein. Two-tailed Mann-Whitney U-test. *p<0.05, n = 5-8. Median is shown. **(F)** Scheme of administration of Ad-9D9 mixed with Ad26-Sars-Cov2 Spike vaccine (Mixed) or administered at separate injection sites, contralateral (Contra). **(G, H)** T cell responses against Sars-Cov2 Spike peptide measured by ex vivo IFN-ɣ ELISpot assay **(G)** and serum anti-Spike antibodies development **(H)**, 14 days after IM injection of Ad26-Spike vaccine alone or in combination with Ad-9D9 given mixed with the vaccine or contralateral to the vaccine. * p <0.05, n = 5-8. Mean ± SEM **(**in **G)** and median **(**in **H)** are shown.

Heavy and light chains DNA sequences of a previously described anti-mouse CTLA-4 monoclonal antibody (clone 9D9, isotype 2a) were cloned in a replication defective Adenovirus 6 (Ad-9D9) (22). We selected the human Adenovirus serotype 6 for its lower seroprevalence compared to human Adeno 5 (23). The layout of the transgene is depicted in **Figure 1A**. Correct expression of 9D9 was shown by infection of HeLa cells with Ad-9D9 and ELISA and Western blot performed on whole cell extracts (**Figure 1B**). Circulating levels of the 9D9 antibody were measured *in vivo* in C57Bl/6 mice by ELISA, at different time points after intramuscular administration of 10^8^ viral particles (vp) of Ad-9D9 or administration of 9D9 as proteinaceous form (**Figure 1C**). The serum levels of Ad-9D9 produced antibody (blue curve) were significantly lower than the antibody levels measured upon administration of the proteinaceous form (red curve), with a delayed peak at 7 days post-injection and no detectable expression by day 14 post-injection. The kinetic of expression, coupled to reduced systemic levels of Adenovector driven anti-CTLA-4, are suggestive of a safer profile of the Ad-9D9 treatment as compared to the canonical administration of the antibody in the protein form.

To test the ability of Ad-9D9 to improve the adaptive immune responses elicited by an Adenovirus-based vaccine, we constructed a replication incompetent Adenovirus serotype 26 expressing the Sars-Cov2 Spike protein (Ad26-Spike). The adjuvant effect of 9D9 on the co-administered Ad26-Spike was evaluated for both the Adeno-encoded and the protein form of the antibody.

We measured the T cell response against a pool of peptides corresponding to the whole Spike protein by *ex vivo* IFN-γ ELISpot in splenocytes of immunized mice (**Figure 1D**), and the development of anti-Spike antibodies in mouse serum (**Figure 1E**), 14 days post immunization. The combined treatment of vaccine Ad26-Spike and Ad-9D9 led to a significant enhancement of T and B cell responses against the Spike antigen as compared to the vaccine alone (~6 fold and 10 fold, respectively; **Figures 1D, E**, blue bars). In contrast, the combination of Ad-Spike with 9D9 in its proteinaceous form resulted in a lower enhancement of both cellular and humoral immune responses, in spite of the larger hematic concentration displayed by the latter form (**Figures 1D, E**, red bars).

To explain this apparent inconsistency, we explored the importance of the co-localization of antigen and antibody at the immunological synapses during the priming phase of the immune response (24). To this aim, we tested two different intramuscular administration modalities of Ad-9D9, as depicted in the scheme in **Figure 1F**: i) co-administered in a mixture with the Ad26-Spike vaccine (Mixed); and ii) injected separately in a contralateral site with respect to the vaccine injection site (Contra). Results of *ex vivo* IFN-γ ELISpot assay showed that only when Ad-9D9 is co-administered with the vaccine as a mixture, the vaccine-induced anti-Spike T cell response is significantly enhanced; conversely, in the case of contralateral administration of vaccine and Ad-9D9, no adjuvant effect was observed (**Figure 1G**, grey bars). Similar results were observed for the induction of anti-Spike antibodies (**Figure 1H**, grey bars).These data indicate that for the Adeno-encoded adjuvant effect to occur, a coordinated expression in space of vaccine antigen and immunomodulator protein is required.

### Ad-9D9 is a potent genetic adjuvant of Adenovirus-based neo-antigen vaccine

To assess whether the use of Ad-9D9 could represent an agnostic technology platform, we tested the ability of Ad-9D9 to enhance the adaptive immunity induced by a different Adenovirus vector encoding a different antigen. To this end, we used a previously described non-human Great Apes Adenovirus (GAd; currently being used in human clinical trials) encoding for seven CD8 neo-antigens selected from the murine colon adenocarcinoma MC38 cell line (GAd-7nag) (25).

**Figure 2:**
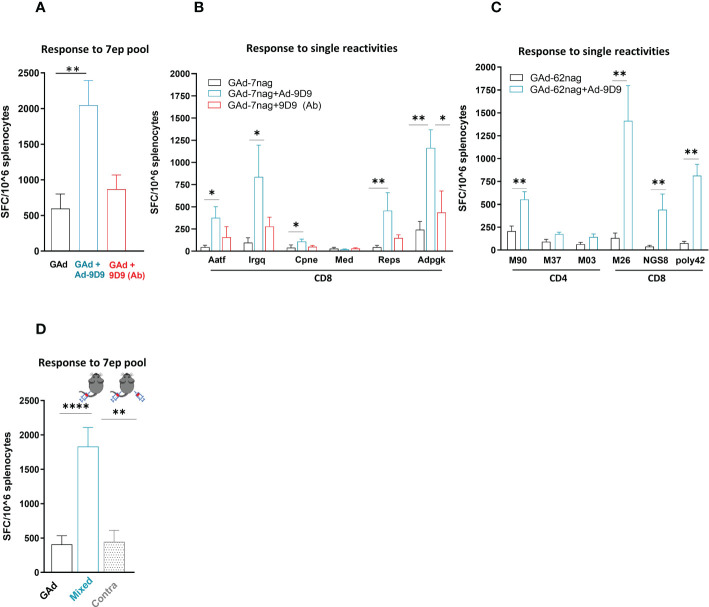
Ad-9D9 has a strong adjuvant effect when co-administered with Ad-based tumor neoantigens vaccine. **(A–C)** Effect of Ad-9D9 versus the 9D9 antibody delivered as protein on the *in vivo* immunogenicity of a GAd vaccine encoding tumor neoantigens selected from murine MC38 (GAd-7nag encoding CD8 neoantigens; **(A, B)** and CT26 tumors (GAd-62nag encoding both CD4 and CD8 neoantigens; **(C)** measured by IFN-γ EliSpot; total response **(A)** or responses against individual nAgs peptides found immunogenic are shown **(B, C)**; MC38 and CT26 neoantigens, respectively) (n = 6 - 10). Two-tailed Mann-Whitney U-test; *=p<0.05; **p=0.004. **(D)** Effect of Ad-9D9 on vaccine-induced T cell responses against the pool of 7 neoantigen peptides measured by IFN-γ EliSpot on mouse splenocytes when the Ad-9D9 injected IM is mixed with the vaccine or administered in two different anatomical sites, contralateral to the vaccine (n = 6 - 10). Two-tailed Mann-Whitney U-test; **p<0.005 ****p<0.0001. MEAN ± SEM is shown. SFC = Spot forming cells.

Co-administration of Ad-9D9 with GAd-7nag in C57Bl/6 mice resulted in a significant improvement of the total T cell responses against the encoded neo-antigens (~4 fold; **Figure 2A**, blue bar). In contrast, the 9D9 in the protein form showed only a modest adjuvant effect compared to the vaccine alone (**Figure 2A**, red bar). The adjuvant effect of Ad-9D9 was observed on five of the six immunogenic neo-antigens, with the most prominent improvements being observed on the weaker T cell reactivities (i.e. Aatf, Irgq with 8 and 9 fold increase, respectively; versus Adpgk with 5 fold, **Figure 2B**). Also in this case, we demonstrated the importance of the co-localization of Ad-9D9 and GAd-7nag to achieve the adjuvant effect on the adaptive immunity (**Figure 2D**). The adjuvant activity of Ad-9D9 was confirmed with a second GAd-based multi-epitope vaccine encoding for 62 CD4 and CD8 neo-antigens selected from the CT26 murine colon carcinoma cell line (GAd-62nag). Co-administration of Ad-9D9 together with the GAd-62nag vaccine in Balb/c mice enhanced both the CD4 and CD8 neo-antigen responses, including the responses to subdominant neo-antigens, such as NGS8 and 62poly42 (**Figure 2C**).

### Ad-9D9 increases the anti-tumor efficacy of GAd-based neoantigen vaccine in combination with anti-PD-1

**Figure 3:**
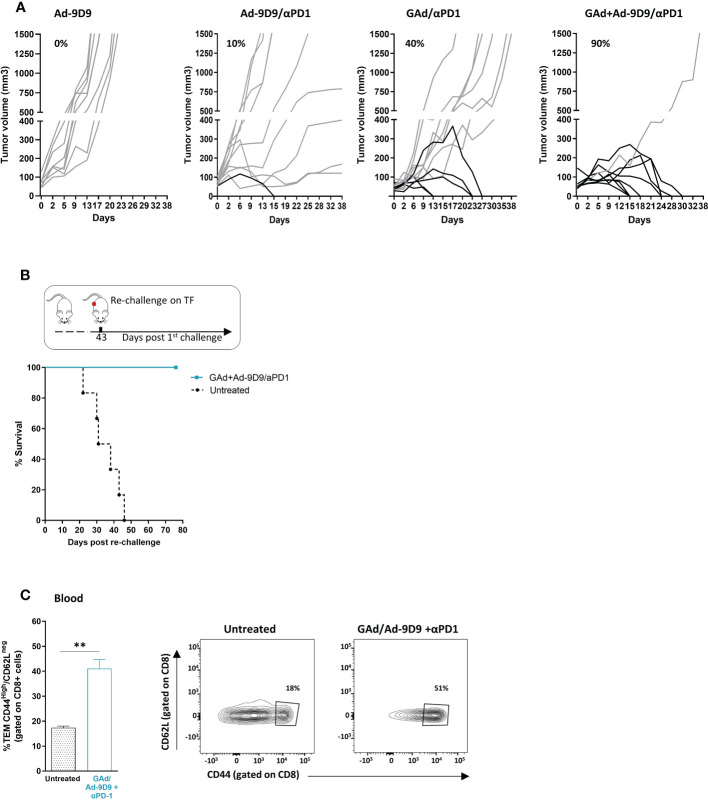
Ad-9D9 increases the anti-tumor efficacy of Ad-based neoantigen vaccine in combination with anti-PD-1. **(A)** Tumor growth measurement (Y axis) over time (days, X axis) in tumor bearing mice (n = 10). Experimental groups are the following: Ad-9D9; combination of Ad-9D9 + anti-PD-1; combination of GAd-62nag vaccine + anti-PD-1; combination of GAd-62nag vaccine + Ad-9D9 + anti-PD-1. Percentages on the graph indicate the frequency of mice showing a complete response (black lines, cured mice). Grey lines indicate not responding mice with growing tumors. **(B)** Tumor free mice from the group treated with GAd-62nag vaccine + Ad-9D9 + anti-PD-1 in **(A)** received a second tumor challenge with CT26 cells on day 43 post the first tumor challenge. Control naïve mice (untreated) were challenged with CT26 (n = 6). Tumor survival was assessed over time. **(C)** Frequency (%) of CD8 effector memory T cells (TEM) identified as CD44^high^/CD62L^low^ as standard phenotype of T effector cells, measured on blood of untreated control mice versus mice receiving the combo of GAd vaccine + Ad-9D9 + anti-PD-1 (n = 3). Two-tailed Mann-Whitney U-test; *p<0.05. Representative FACS plots are shown.

In murine models, CTLA-4 blockade has been demonstrated to enhance T cell responses to cancer vaccines and tumor infiltration along with intratumoral Treg depletion, resulting in tumor control and clearance (18, 26). To determine whether Ad-9D9 could improve anti-tumoral efficacy of a neo-antigen based vaccine, we tested its administration in combination with a GAd-62nag and anti-PD-1 immunotherapy, in a CT26-established tumor model in Balb/c mice (**Figure 3A**). In line with our previously data, vaccine and anti-PD-1 induced anti-tumor response in 40% of treated mice which underwent complete rejection of large tumors (21). Co-administration of Ad-9D9 with GAd-62nag and anti-PD-1 increased the rate of tumor regression to 90%. Ad-9D9 alone or in combination with anti-PD-1 resulted in very poor anti-tumor efficacy (**Figure 3A**). Anti-tumor efficacy was further confirmed in a second tumor model, the MC38 colon adenocarcinoma, where the triple combination (GAd vaccine, Ad-9D9 and anti-PD-1) resulted in the complete tumor eradication in 90% of animals, whereas the rate of cure decreased to 37% and 0%, without the vaccine or without anti-PD-1, respectively (**Supplementary Figure 1**). Mice that became tumor free after the treatment with the triple combination with GAd vaccine, Ad-9D9 and anti-PD-1 were all protected from a second CT26 tumor challenge (**Figure 3B**). An increase in circulating effector/memory CD8 T lymphocytes was also observed after the same treatment, suggesting the generation of systemic protective immune memory (**Figure 3C**).

### The combination of Ad-9D9 with GAd vaccine and anti-PD-1 reshapes the tumor microenvironment by expanding CD8+ T cells and reducing intratumoral Tregs cells

**Figure 4:**
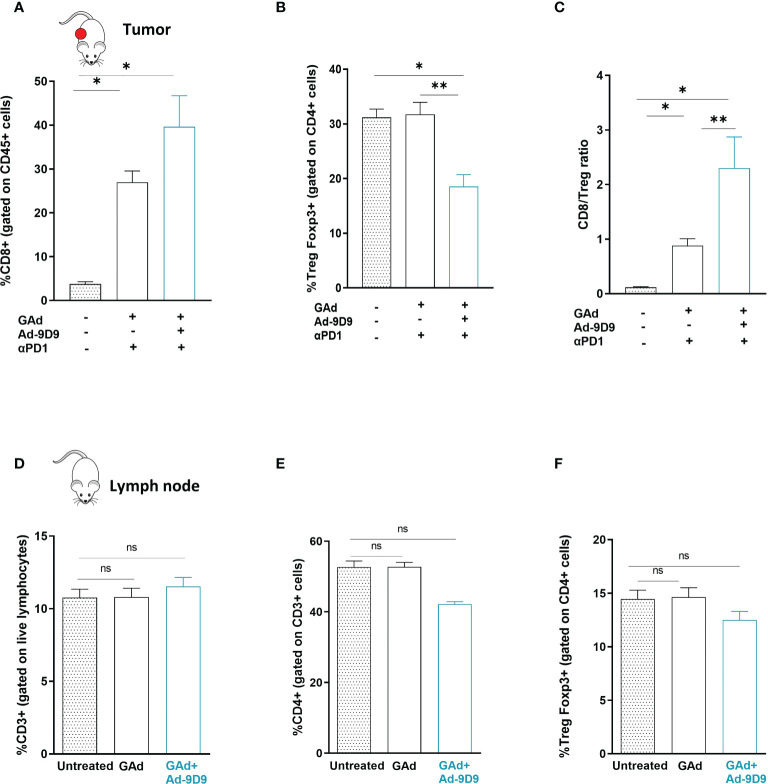
Combination of Ad-9D9 with GAd vaccine and anti-PD-1 reshapes the tumor microenvironment by reducing intratumoral Tregs and expanding CD8+ T cells **(A–C)** Flow cytometry analysis of tumor infiltrating lymphocytes cells from CT26 tumors collected at day 9 post treatment with GAd-62nag vaccine + anti-PD-1, with or without Ad-9D9 compared to untreated tumors. **(A)** CD8+ T cells were gated on live CD45+ lymphocytes (n = 3); **(B)** Intratumoral Tregs were Foxp3+ cells gated on CD4+ cells. Two-tailed Mann-Whitney U-test; *p<0.05. **(C)** Intratumoral CD8+/Treg ratio. Two-tailed Mann-Whitney U-test; *p<0.05; **p<0.005; ns, not significant. **(D–F)** Peripheral immune T cells isolated from iliac lymph nodes of naïve non-tumor bearing mice do not change upon Ad-9D9 treatment.

Cytofluorimetric analysis of the tumor immune infiltrates showed that inclusion of Ad-9D9 to the combination of GAd-62nag and anti-PD-1 induces the highest increase in CD8 T cells frequency and the deepest depletion of intratumoral Treg cells, compared with the GAd-62nag vaccine alone and the dual combination GAd-62nag + anti-PD-1 (**Figures 4A, B**). These changes in the tumor infiltrate resulted in the highest CD8/Treg ratio (**Figure 4C**), correlating with the best rate of anti-tumor efficacy observed in the group that received the triple combination GAd-62nag, anti-PD-1 and Ad-9D9. No changes in peripheral immune populations in iliac lymph nodes were observed (**Figures 4D–F**), indicating that the reshaping of the immune microenvironment induced by Ad-9D9 is specific for the tumor immune infiltrate.

### Encoded human Ipilimumab enhances the vaccine induced T cell responses

**Figure 5:**
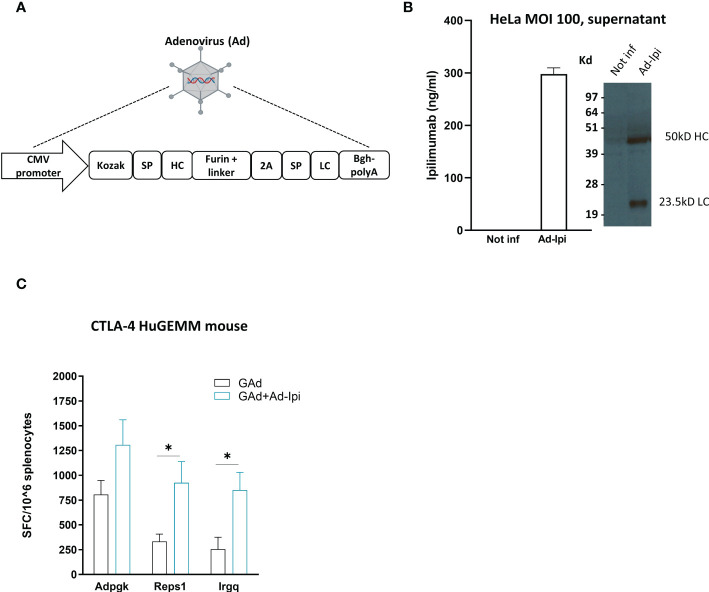
*In vivo* adjuvant effect of Adeno-encoded human anti-CTLA-4 on vaccine induced T cell responses **(A)** Layout of Ipilimumab construct encoded in a replication defective Adenovirus 6 (Ad-Ipi). SP = Signal peptide; HC = Heavy chain; LC = Light chain. **(B)** *In vitro* expression of encoded Ipilimumab in HeLa cells infected with Ad-Ipi at the indicated MOI (Multiplicity of infection) and assessed by Elisa and Western Blot on supernatant collected 48 hours post infection. *In vitro* experiments were performed in triplicate. **(C)** Effect of Ad-Ipi on immunogenicity of GAd-7nag vaccine in transgenic mice expressing human CTLA-4 (CTLA-4 huGEMM). T cell responses against 3 individual neoantigens (Adpgk, Reps1, Irgq) were measured by IFN-γ EliSpot on mouse splenocytes (n = 6), 14 days after vaccination with GAd-7nag vaccine administered alone or in combination with Ad-Ipi. Two-tailed Mann-Whitney U-test; *p<0.05.

An encoded version of human anti-CTLA-4 monoclonal antibody Ipilimumab (isotype IgG1) was generated in a replication defective Adenoviral vector 6 (Ad-Ipi, **Figure 5A**). HeLa cells infected with Ad-Ipi were shown to express and secrete human anti-CTLA-4, as detected by Elisa and Western Blot on supernatant collected 48 hours post-infection (**Figure 5B**).

To investigate whether Ad-Ipi was functional as immunomodulator of virus vectored genetic vaccines -induced adaptive response, we used a transgenic C57Bl/6 mouse strain expressing human CTLA-4 (CTLA-4 huGEMM mice). Combination of Ad-Ipi with Ad-7nag vaccine enhanced the antigen-specific T cell immune response (**Figure 5C**), confirming that also the encoded version of the human anti-CTLA-4 antibody increased the vaccine-induced T cell responses similar to what was observed for the murine version of the encoded antibody.

## Discussion

Virus vectored genetic vaccines offer the advantage over traditional vaccines, to induce both B- and T-cell responses. In particular, replication incompetent Adenovirus vectors have been shown to achieve high levels of CD8 cytotoxic T lymphocytes besides CD4 and neutralizing antibodies, leading to the development of commercially approved Sars-Cov2 vaccines, and are currently being tested in the clinic for the development of a new generation of therapeutic vaccines based on tumor neo-antigens (27, 28). However, further improvement of vaccine potency is always desirable in many different circumstances. For example, during the development of the Adenovirus-based COVID-19 vaccines *INN-Ad26.COV2-S* and *Vaxzevria*, it was noted that the level of vaccine-induced virus neutralizing antibodies was lower than those induced by mRNA vaccines or recombinant protein-based vaccines, and a booster dose was shown to be needed to improve potency and durability of the immune response. A possible solution to these limitations would be to use adjuvants. However, since most known adjuvants (Alum, MF59, ASO4 etc.) activate innate immunity, and do so very soon after administration, it is to be expected that their use in combination with Adenovirus based vaccines would hamper the expression of the encoded antigen, thereby having the opposite effect and reducing vaccine immunogenicity. Therefore, new modalities of vaccine adjuvantation are needed for Adenovirus vectored vaccines, where the presence of antigen and adjuvant should be ‘synchronized’ in time and space. Here, we employed the approach of genetic adjuvants as alternative to the “conventional” adjuvants, by using encoded immunomodulatory molecules co-delivered with the genetic vaccine vector, allowing for a local and timely expression of the immunomodulator together with the antigen. We generated an Adeno vector encoding for a murine anti-CTLA-4 monoclonal antibody (Ad-9D9) to be used as a genetic adjuvant to improve adaptive immunity and efficacy of Adeno based vaccines. To test this hypothesis we chose the Adenovector/antigen combination of a replication incompetent human Adenovirus serotype 26, the same used for the approved anti-COVID-19 vaccines INN- Ad26.COV2-S, encoding for the full length Sars-Cov2 Spike glycoprotein (Ad26-Spike). We demonstrated that the combination of encoded anti-CTLA-4 with Ad26-Spike induced a significant enhancement of T cell response against the Spike antigen, as well as a higher titer of anti-Spike antibodies as compared to the Ad26-Spike vaccine alone. The effect of anti-CTLA-4 on antibody response has been already demonstrated *via* a T-cell dependent effect (20), therefore a possible mechanism behind enhanced anti-Spike Ab response could be related to the enhancement of CD4 T cell response which can provide “help” for antibody production by B cells. Interestingly, anti-CTLA-4 in its protein form did not achieve the same potent adjuvant effect in spite of the much higher levels of antibodies measured in the blood of immunized animals. One possible explanation for this difference is that the use of the same delivery vectors to encode the antigen and the immunomodulatory molecule, when co-delivered as mixture, increases the likelihood that both the antigen and the immunomodulator will be expressed at the same site and with a similar kinetic for a more effective generation of the antigen-specific immune response. This hypothesis is corroborated by the observation that the adjuvant effect of Ad-9D9 was lost when it was administered contralateral with respect to vaccine injection site. The importance of the synchronism between Adeno-encoded adjuvant and Adeno-based vaccine, not only in space but also in time was demonstrated by the loss of effect when Ad-9D9 was administered delayed in time respect to the vaccine (data not shown).

The AdEnA approach may represent a technology platform, offering the advantage of flexibility to combine the adjuvant-expressing vector with a number of existing Adeno-based clinical vaccines, for various indications, since the Ad-9D9 adjuvant effect on Adenovirus vectored vaccine is antigen and vector independent. Accordingly, the activity of the Adeno encoded anti-CTLA-4 was confirmed when it was used in combination with a different Adenovirus vaccine vector (GAd) encoding cancer neo-antigens, whereby it broadened the repertoire of tumor-specific T cells, by enhancing the immune responses to sub-dominant epitopes and leading to better poly-specific anti-tumor immunity. The increase of epitope breadth is an important aspect in the context of tumor-specific T cell responses. “Ignored” (29) neo-antigens often do not induce a relevant immune response likely because the level of presentation is below the threshold for priming of naïve T cells. However, they may contribute as effective epitopes to protective immunity. Moreover, the increase of epitope specific response potentially reduces the risk of tumor escape through immune-editing. Also in this case, the encoded immunomodulator achieved higher immunological effect as compared to its proteinaceous form when tested in combination with the vaccine, further underlying the advantage of delivering the encoded immunomodulator together with the vaccine, both as Adenoviral format, directly at the priming sites, where T cell activation occurs. In parallel with the improved vaccine immunogenicity, the delivery of the encoded anti-CTLA-4 in combination with an Adeno neo-antigen based vaccine and anti-PD-1 antibody led to almost 100% cure of large established tumors in mice, with a significant improvement of anti-tumoral activity as compared to the dual combination based on the vaccine and anti-PD-1. Anti-CTLA-4 antibody can have itself strong activity in murine models, as shown in Duperret et al. (22). However, the very high rate of response observed in their work with monotherapy ani-CTLA4 may due to the different experimental settings used. In Duperret et al., anti-CTLA-4 antibody is administered in an early therapeutic setting, whereas in our work, we used a setting of advance therapeutic treatment. In the very same model, we have previously demonstrated how different tumor settings impact on the effectiveness of the immunotherapy, with 100% of therapeutic response with an Adenovirus based vaccine encoding cancer-derived neoantigens in CT26 early setting, and lack of any effect of the same vaccine in advanced setting, unless combining the vaccine with anti-PD1 (21). The addition of encoded anti-CTLA-4 to the prior tested dual therapy of GAd neo-antigen vaccine and anti-PD-1 (21) resulted in further increased levels of CD8 T cell infiltration at the tumor site and a concomitant decrease of the immunosuppressive Treg population with a shift in the intratumoral balance from an immunosuppressive to a permissive state, in line with the known ability of anti-CTLA-4 to modulate effector CD8 T cells and Treg cells in the tumor (18). Interestingly, the reduction of Treg cells was only found in the tumor, and not in the periphery, suggesting that the enhancement of vaccine induced immune responses measured in the periphery is independent from the depleting effect on Treg cells occurring in the tumor. The efficacy of Ad-9D9 in controlling tumor growth in association with a vaccine and anti-PD-1 is due to i) the depletion of intratumoral Tregs and the increase of intratumoral T cell effectors; ii) the enhancement of vaccine-induced immune responses with memory function. Importantly, all these effects were obtained with negligible amounts of antibody released in the circulation and without any sign of discomfort in treated mice, minimizing the risk of toxicity without compromising the adjuvant and the anti-tumor effects of anti-CTLA-4. The observation that the encoded version of the anti-CTLA-4 antibody was more potent than its proteinaceous counterpart, despite its reduced systemic exposure, becomes particularly relevant when considering the autoimmune toxicity associated with the administration of anti-CTLA-4 antibody in patients. Our findings pave the way to the development of a novel class of adjuvants for virus vectored genetic vaccines, not only for the higher potency displayed by the vectored immunomodulator with respect to its proteinaceous form, but also because Adenovirus vectors have been shown to be immunogenic, safe and efficacious in humans and can be produced at a commercial scale and with highly competitive costs, making feasible the rapid translation of the AdEnA approach into the clinic.

## Data availability statement

The raw data supporting the conclusions of this article will be made available by the authors, without undue reservation.

## Ethics statement

The animal study was reviewed and approved by Italian Ministry of Health.

## Author contributions

ESc, AN, and AMD’A designed the study. AMD’A, LN, IG, LS, LI, FT, GC, SA, GR, GL, ESa conducted the research. AMD’A, LN, ESc, AN analyzed and interpreted the data. AL contributed to data interpretation. AMD’A, LN, AN, ESc wrote the manuscript. ESc and AN were the principal investigator and act as guarantors. All authors contributed to the article and approved the submitted version.
