# Supplementary material for: Adenovirus Encoded Adjuvant (AdEnA) anti-CTLA-4, a novel strategy to improve Adenovirus based vaccines against infectious diseases and cancer
Source: Front Immunol. 2023 Apr 26;14:1156714. doi: 10.3389/fimmu.2023.1156714 (PMC10169702; doi:10.3389/fimmu.2023.1156714)
Supplement: Supplementary file 1 [file DataSheet_1.docx]

Supplementary Material

**Adenovirus Encoded Adjuvant (AdEnA) anti-CTLA-4, a novel strategy to improve Adenovirus based vaccines against infectious diseases and cancer**

**Anna Morena D’Alise* , Linda Nocchi, Irene Garzia , Laura Seclì, Luigia Infante , Fulvia Troise, Gabriella Cotugno, Simona Allocca, Giuseppina Romano, Armin Lahm, Guido Leoni, Emanuele Sasso, Elisa Scarselli and Alfredo Nicosia**

*** Correspondence:** Corresponding Author: [**m.dalise@nouscom.com**](mailto:m.dalise@nouscom.com)

# Supplementary Data

Complete amino acid sequence of 9D9 encoded

**MGWSWIFLFLLSGTAGVLSEVQLQQSGPVLVKPGASVKMSCKASGYTFTDYYMNWVKQSHGKSLEWIGVINPYNGDTSYNQKFKGKATLTVDKSSSTAYMELNSLTSEDSAVYYCARYYGSWFAYWGQGTLVTVSS**AKTTAPSVYPLAPVCGDTTGSSVTLGCLVKGYFPEPVTLTWNSGSLSSGVHTFPAVLQSDLYTLSSSVTVTSSTWPSQSITCNVAHPASSTKVDKKIEPRGPTIKPCPPCKCPAPNLLGGPSVFIFPPKIKDVLMISLSPIVTCVVVDVSEDDPDVQISWFVNNVEVHTAQTQTHREDYNSTLRVVSALPIQHQDWMSGKEFKCKVNNKDLPAPIERTISKPKGSVRAPQVYVLPPPEEEMTKKQVTLTCMVTDFMPEDIYVEWTNNGKTELNYKNTEPVLDSDGSYFMYSKLRVEKKNWVERNSYSCSVVHEGLHNHHTTKSFSRTPGKRGRKRRS***GSG*APVKQTLNFDLLKLAGDVESNPGPMKLPVRLLVLMFWIPASSSDIVMTQTTLSLPVSLGDQASISCRSSQSIVHSNGNTYLEWYLQKPGQSPKLLIYKVSNRFSGVPDRFSGSGSGTDFTLKISRVEAEDLGVYYCFQGSHVPYTFGGGTKLEIKR**ADAAPTVSIFPPSSEQLTSGGASVVCFLNNFYPKDINVKWKIDGSERQNGVLNSWTDQDSKDSTYSMSSTLTLTKDEYERHNSYTCEATHKTSTSPIVKSFNRNEC

**Amino acid sequence of murine anti-CTLA-4 9D9.** The complete amino acid sequence of 9D9 heavy and light chain variable domains was extracted from published sequences (US9868961B2). The sequence of the heavy chain variable domain sequence was modified according to “mod #4”, as described in (22). The sequence was assembled as: 9D9 VH signal sequence (bold black) followed by the 9D9 VH domain (bold red); then the mouse IgG2A heavy chain constant region (red underlined) followed by a RGRKRRS cleavage site (black underlined) and a SGS linker (bold black italics), followed by a P2A peptide sequence (bold green), a mouse kappa light chain signal sequence (bold brown), the 9D9 VL domain (bold blue) and the mouse kappa light chain constant domain (blue underlined).

# Supplementary Figure 1


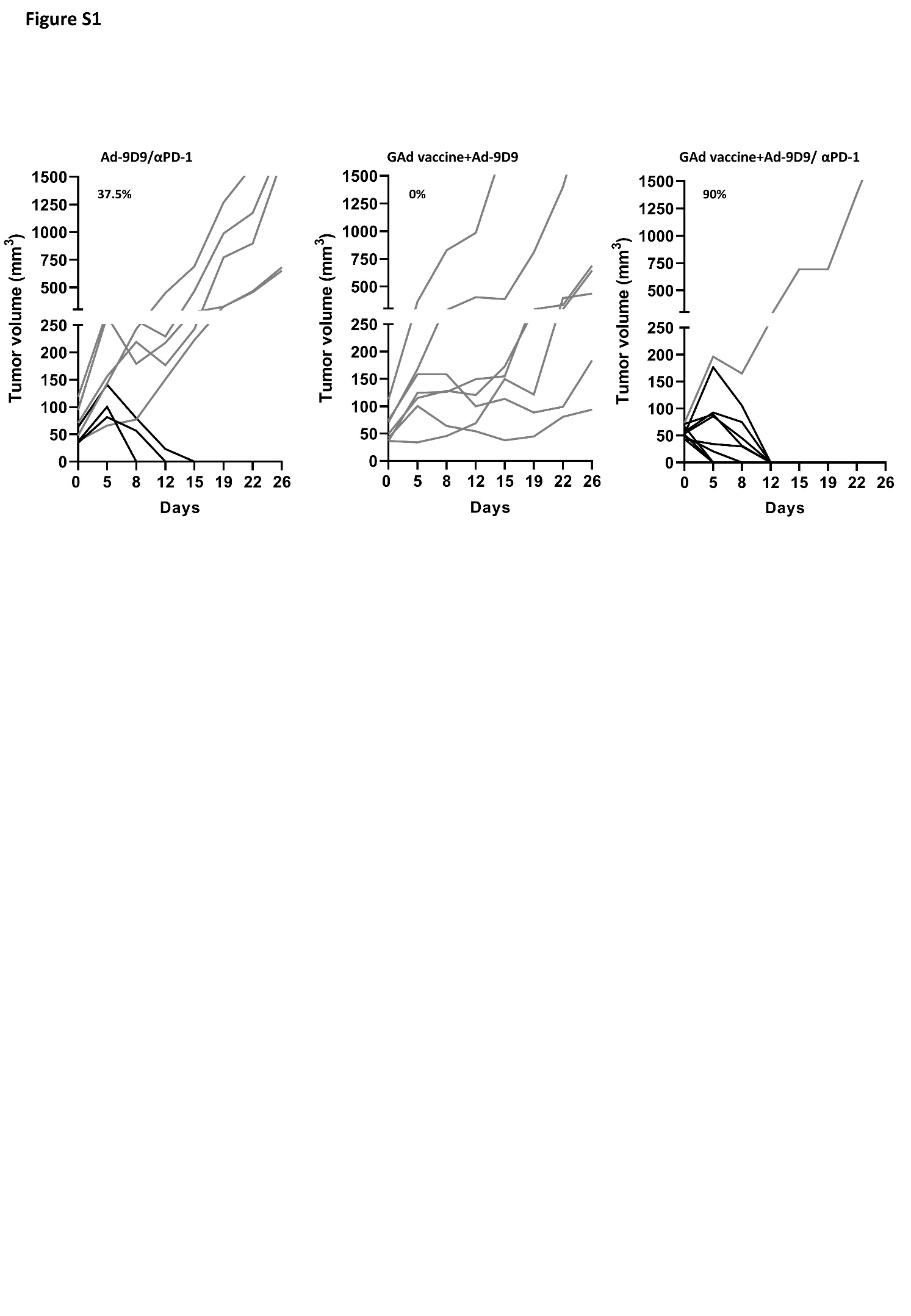


**Supplementary Figure 1.** **Anti-tumor activity of Ad-9D9 in combination with a GAd neoantigen-based vaccine and anti-PD-1 in MC38 established tumor model.** Tumor growth in MC38 tumor bearing mice (n = 7-10) treated with Ad-9D9/aPD-1 or GAd-MC38-32ep vaccine + Ad-9D9 or in triple combination GAd-MC38-32ep vaccine + Ad-9D9 + anti-PD-1. Percentages on the graph indicate the frequency of mice showing a complete response (black lines, cured mice). Grey lines indicate not responding mice with growing tumors.

.
